# Exploring trends and determinants of basic childhood vaccination coverage: Empirical evidence over 41 years

**DOI:** 10.1371/journal.pone.0300404

**Published:** 2024-03-21

**Authors:** Paloma Lanza-León, David Cantarero-Prieto, Marta Pascual-Sáez

**Affiliations:** 1 Departamento de Economía, Universidad de Cantabria, Santander, Spain; 2 Health Economics Research Group, Valdecilla Biomedical Research Institute—IDIVAL Santander, Spain; 3 Santander Financial Institute—SANFI, Santander, Spain; King Faisal University, SAUDI ARABIA

## Abstract

Vaccination is widely considered to be one of the most important prevention measures as a health strategy. This paper examines trends in basic childhood vaccination coverage and which country and time-dependent determinants may have influenced childhood immunization rates (1-dose BCG, 1- and 3-dose DTP (diphtheria, tetanus, pertussis), 1-dose measles, and 3-dose polio) between 1980 and 2020 across 94 countries. We identify economic, inequality, demographic, health, education, labor market, environmental, and political stability factors of immunization. To do this, we use data from the annual WHO and United Nations International Children’s Emergency Fund (UNICEF) coverage estimates. The empirical analysis consists of generalized estimating equation models to assess relationships between immunization rates and socioeconomic factors. Additionally, we follow the Barro and Sala-i-Martín approach to identify conditional convergence. Our findings show the strongest positive statistically significant association between immunization rates and GDP per capita, as well as births attended by skilled health staff. Moreover, our research demonstrates conditional convergence, indicating that countries converge towards different steady states. The present study brings new insights to investigating the determinants of childhood vaccination coverage and provides significant implications for health policies.

## Introduction

Childhood vaccination is widely considered to be one of the most important prevention measures as a health strategy and one of the major public health interventions for improving the population’s health and well-being. However, the benefits of vaccines go beyond prevention, providing economic and social benefits to individuals and society [1]. In other words, childhood vaccination achieves high savings in healthcare costs and avoids productivity losses, as well as improving cognitive development, educational performance, health equity and social integration [2].

Improving global childhood vaccination coverage prevents life-threatening diseases and avoids 3.5–5 million deaths every year worldwide. However, this is only possible when vaccines reach the intended target populations. The World Health Organization (WHO) has set several targets to achieve childhood vaccination rates of 95% [3]. Since the creation of the Global Alliance for Vaccines and Immunizations (Gavi), also referred to as the Vaccine Alliance, in 2000, there have been improvements in health and development, with an increase in the number of routine introductions, prevention campaigns, and vaccination coverage in low- and middle-income countries, especially in the world’s poorest ones [4]. In addition, the objectives of the Global Immunization Agenda 2030 include equitable immunization coverage between and within countries, dealing with disadvantages by gender, age, and other socioeconomic characteristics [5]. However, there is still a need to improve childhood immunization rates for the most disadvantaged groups, such as children from low-income countries, those from gypsy traveler communities and children who have been in the care of the child welfare systems [6–8]. In other words, if low routine immunization coverage persists (partial vaccination as well as non-vaccination), children are more likely to become ill, disabled or even have a higher risk of morbidity and mortality [9, 10]. Vaccination can help prevent and avoid infections and, consequently, improve health outcomes, schooling, and productivity in adulthood [11, 12].

Previous scientific research has summarized determinants associated with the uptake of routine vaccines which threaten global health, both on the supply and demand side [13–17]. Some of these factors may include immunization programs, services, stock-outs, logistics and health infrastructures, as well as the perception of adverse effects, knowledge, beliefs, hesitancies, social influences and other general parental and family attitudes towards children [18, 19]. Furthermore, studies analyzing low- and middle-income countries have identified low income, lack of access to health care, low parental education and unassisted childbirth as risk factors for low vaccination rates [20–22]. All these factors listed above could lead to higher or lower (under-immunization) rates of childhood vaccination, especially in certain population subgroups. In this context, immunization rates differ significantly between countries with completely different income levels.

Most of the existing literature on this topic has analyzed determinants associated with basic childhood immunization either at an individual or local level within a country, focusing mainly on a single low- or middle-income country [23–26]. In addition, literature is also limited to a single vaccine [27–30].

As mentioned above, the main objective of this study is to analyze trends in childhood immunization coverage (1-dose BCG, 1- and 3-dose DTP (diphtheria, tetanus, pertussis), 1-dose measles, and 3-dose polio) and a range of factors (economic, inequality, demographic, health, education, labor market, environmental, and political stability) at a national level, which could be associated with basic vaccination rates. The rationale for tracking certain socioeconomic factors that have influenced immunization coverage over a 41-year period is to shed light on the role these determinants could play in vaccination across different countries and to identify key areas of intervention to help vaccine program managers and policymakers. As an additional objective, this study also asks whether a conditional convergence of vaccination coverage rates exists. The rationale for analyzing convergence is to test whether the countries considered, which have similar structural parameters, behave similarly to each other. To do so, we examine as many countries and vaccines as possible, taking into consideration the availability of data from the World Bank and WHO for the period between 1980 and 2020. As far as we are aware, this study provides the most recent evidence on country-level predictors in basic childhood vaccination across 94 countries. We have focused on five vaccines included in the global immunization schedules and a range of socioeconomic determinants that may influence childhood vaccination rates, showing differences between low-, lower-middle-, upper-middle- and high-income countries.

## Materials and methods

### Data sources

With the first objective of this study in mind, i.e., analyzing trends in childhood immunization coverage, we considered data from five vaccines, which are listed below, based on the data available for 94 countries worldwide over the past four decades. We considered a time horizon (from 1980 to 2020) that was long enough for analyzing trends in vaccination and relied on data from the World Bank to identify the determinants of childhood vaccination coverage. Finally, we took as large a sample as possible into account when it came to selecting countries, always based on the availability of data.

The outcome variables in this study are the country’s vaccination coverage of the first dose of the Bacillus Calmette-Guérin (BCG) vaccine, the first and the third dose Diphtheria, Pertussis and Tetanus vaccine (DTP1 and DTP3), the first dose of the Measles-Containing vaccine (MCV1), and the third dose of the Polio vaccine (Pol3). National vaccine coverage was used as an indicator for childhood immunization programs. We used data from the annual WHO and United Nations International Children’s Emergency Fund (UNICEF) coverage estimates [31] for these five vaccines for the 41-year period analyzed (i.e., 1980–2020). The summary statistics for the outcome variables in 1980 and 2020 are shown in Table 1. We observe how the average coverage rates of all considered vaccines increase during the analyzed 41-year period. Additionally, the standard deviation among countries decreases at the end of the period.

**Table 1:** Summary statistics for the outcome variables for 1980 and 2020.

Dependent variables	Obs.	Coverage (%)	Std. Dev.	[95% conf. interval]	
1980					
BCG	65	55.60	3.85	47.90	63.30
DTP1	92	66.46	2.56	61.38	71.53
DTP3	92	47.55	3.06	41.47	53.64
MCV1	60	43.02	3.59	35.83	50.21
Pol3	89	48.81	3.43	41.99	55.62
2020					
BCG	79	84.35	2.38	79.60	89.08
DTP1	94	90.47	1.16	88.16	92.77
DTP3	94	86.09	1.45	83.20	88.97
MCV1	94	86.28	1.38	83.54	89.01
Pol3	94	85.47	1.43	82.62	88.32

Note: Bacillus Calmette-Guérin (BCG) Diphtheria, Pertussis and Tetanus (DTP); Measles-Containing vaccine (MCV); Polio (Pol3). Source: compiled by the author.

The selection of independent variables used in this study was motivated by previous literature [14, 32–34] and the availability of data in the World Bank and WHO datasets. We considered 23 characteristics in the analysis related to economic, inequality, demographic, health, education, labor market, environmental, and political stability determinants to identify which indicators might be related to childhood immunization coverage. Economic factors covered Gross Domestic Product (GDP) per capita and current health expenditure. The Gini index was used to measure inequality. The demographic determinants involved were total population, population aged between 15 and 65, population aged 65 and over, population growth and urban population. Moreover, health characteristics were made up of crude birth rates, births attended by skilled health staff, hospital beds, the number of nurses/midwives and physicians, and the share of pregnant women receiving prenatal care. We included the literacy rate as the education factor analyzed. The labor predictor considered was the unemployment rate. The environmental indicator analyzed was the country’s surface area (country’s total area measured in square kilometers). Finally, as far as determinants of political stability were concerned, in the analysis we included the Worldwide Governance Indicators for voice and accountability, political stability and absence of violence/terrorism, government effectiveness, regulatory quality, rule of law and control of corruption. The estimates for determinants of political stability range from approximately -2.5 (weak) to 2.5 (strong) governance performance. The list of initial covariates and their description is shown in Table 2.

**Table 2:** List of initial covariates and summary statistics for 2020 or last year available.

Category	Independent variables	Description	Mean	Std. Dev.
*Economic*	GDP per capita (current international $)	Gross domestic product divided by midyear population, measured in current international dollars.	9.58	1.05
	Current health expenditure (% of GDP)	Level of current health expenditure expressed as a percentage of GDP.	6.77	3.24
*Inequality*	Gini index	The extent to which the distribution of income among individuals or households within an economy deviates from a perfectly equal distribution.	44.46	4.87
*Demographic*	Population, total	All residents regardless of legal status or citizenship.	15.74	2.20
	Population aged 15–64 (% of total)	Total population between the ages of 15 and 64 as a percentage of the total population.	65.00	5.76
	Population aged 65 and over (% of total)	Population aged 65 and over as a percentage of the total population.	9.07	6.22
	Population growth (annual %)	Annual percentage growth rate of the population.	1.01	1.00
	Urban population (% of total)	The percentage of people living in urban areas.	62.17	23.37
*Health*	Crude birth rate (per 1,000 people)	Number of live births occurring during the year, per 1,000 people.	17.85	8.04
	Births attended by skilled health staff (% of total)	The percentage of deliveries attended by personnel trained to give the necessary supervision, care, and advice to women during pregnancy, labor, and the postpartum period, to conduct deliveries on their own, and to care for newborns.	96.37	7.38
	Hospital beds (per 1,000 people)	Inpatient beds available in public, private, general, and specialized hospitals, and rehabilitation centers, per 1,000 people.	2.57	2.24
	Nurses and midwives (per 1,000 people)	Professional nurses, professional midwives, auxiliary nurses, auxiliary midwives, enrolled nurses, enrolled midwives, and other associated personnel, per 1,000 people.	4.71	3.98
	Physicians (per 1,000 people)	General and specialist medical practitioners, per 1,000 people.	1.95	1.41
	Share of pregnant women receiving prenatal care (%)	The percentage of women attended at least once during pregnancy by skilled health personnel for reasons related to their pregnancy.	94.52	3.07
*Education*	Total adult literacy rate (% of people aged 15 years and over)	The percentage of people aged 15 and over who can both read and write, and understand a short simple statement about their everyday lives.	88.70	16.80
*Labor*	Unemployment rate (% of total labor force)	The share of the labor force that is without work but available for and seeking employment.	8.52	5.57
*Environment*	Surface area (sq. km)	Country’s total area measured in square kilometers.	11.46	2.63
*Political and institutional*	Voice and accountability	The extent to which a country’s citizens can participate in choosing their government, freedom of expression, freedom of association and a free media.	0.07	0.99
	Political stability and absence of violence/terrorism	The likelihood of political instability and/or politically motivated violence, including terrorism.	0.00	1.04
	Government effectiveness	The quality of public services, the quality of the civil service and the degree of its independence from political pressures, the quality of policy formulation and implementation, and the credibility of the government’s commitment.	0.04	0.98
	Regulatory quality	The ability of the government to formulate and implement sound policies and regulations that permit and promote private sector development.	0.02	0.99
	Rule of law	The extent to which agents have confidence in and abide by the rules of society.	0.07	1.00
	Control of corruption	The extent to which public power is exercised for private gain.	0.08	1.02

Note: GDP (Gross Domestic Product). Source: compiled by the author.

Countries are divided into four groups according to the World Bank’s classification by income level. Firstly, low-income economies are those countries with a Gross National Income (GNI) per capita lower than $1,085. Secondly, lower-middle income economies are countries whose GNI per capita is between $1,086 and $4,255. Thirdly, upper-middle income economies have a GNI per capita between $4,256 and $13,205. Lastly, high-income economies are those countries with a GNI per capita of $13,205 or more. Consequently, we are able to classify countries into these four categories. As per the World Bank’s classification, the 94 countries in our final sample were broken down as follows: 8 low-income economies, 23 lower-middle income countries, 33 upper-middle income economies, and 30 high-income countries (Table 3). This selection and classification allowed us to take a comprehensive approach to the different determinants considered in order to observe the differences between countries.

**Table 3:** Countries classified by Gross National Income per capita (2021).

**Low-income economies**			
Central African Republic	Ethiopia	Somalia	Syrian Arab Republic
Korea, Dem. People’s Rep.	Malawi	Sudan (the)	Yemen, Rep.
**Lower-middle income economies**			
Bhutan	Haiti	Mongolia	Philippines (the)
Bolivia	Honduras	Myanmar	Samoa
Congo, Rep.	India	Nepal	Solomon Islands
Egypt, Arab Rep.	Iran, Islamic Rep.	Nicaragua	Sri Lanka
El Salvador	Kiribati	Pakistan	Tanzania
Ghana	Lesotho	Papua New Guinea	
**Upper-middle income economies**			
Albania	Dominica	Jamaica	Peru
Argentina	Dominican Republic	Jordan	Saint Lucia
Belize	Ecuador	Libya	Saint Vincent and the Grenadines
Botswana	Fiji	Malaysia	Suriname
Brazil	Grenada	Maldives	Thailand
Bulgaria	Guatemala	Mauritius	Tonga
Colombia	Guyana	Mexico	Turkey
Costa Rica	Iraq	Paraguay	Tuvalu
Cuba			
**High-income economies**			
Australia	Denmark	Netherlands (the)	Saint Kitts and Nevis
Austria	Finland	New Zealand	Saudi Arabia
Bahamas, The	Greece	Oman	Sweden
Bahrain	Hungary	Panama	United Arab Emirates
Barbados	Iceland	Poland	United Kingdom of Great Britain and Northern Ireland
Belgium	Ireland	Portugal	United States of America (the)
Brunei Darussalam	Israel	Qatar	Uruguay
Chile	Japan		

Note: GNI per capita lower than $1,085 (low-income economies); GNI per capita between $1,086 and $4,255 (lower-middle income economies); GNI per capita between $4,256 and $13,205 (upper-middle income economies); GNI per capita of $13,205 or more (high-income economies). Source: Compiled by the author using data from the World Bank (2022).

### Statistical analysis

Data from the World Bank and the WHO were pooled to assess trends of vaccination coverage and to examine the socioeconomic factors related to childhood immunization for 94 countries, over a 41-year period (1980–2020). Our initial dataset consisted of five dependent and 23 exogenous variables. However, we employed a machine learning algorithm, in this case the random forests regression model, as a variable-selection technique, following Genuer et al. [35]. We performed the random forests classification for factor selection in order to determine the most important independent variables. The random forest technique builds a set of decision trees. The tree training is performed using only a part of the original data and the rest of the data is held for model validation. Meanwhile, the split-variable for each split on each tree is chosen from a random sample of the initial independent variables. Furthermore, we also identified the top determinants of vaccination coverage rates among the 23 initial independent variables for constructing the final regression model. We checked the variable importance indicator and counted how many times each covariate appeared among the "top 20" variable importance scores. Based on the variable importance, our covariates were reduced by slightly more than 50%. In other words, the analysis was carried out taking 11 variables into consideration. After selecting the variables, the missing data was 32% of a total of 42,394 observations (41 years considered for 94 countries for 11 variables). Using the smaller sample, we performed the imputation of missing data by iterating over a conditionally specified imputation model for each incomplete variable. The algorithm takes into account the available features for that specific case and considers a random subset of features for splitting at each node. Even though values are missing for some of the features considered in our analysis, the algorithm can make decisions based on the available features.

In addition, Generalized Estimating Equations (GEE) were performed to determine the association between basic vaccination coverage and economic, inequality, demographic, health, education, labor market, environmental, and political stability determinants. GEE is a method commonly used to analyze data when several important independent variables are taken into consideration [36]. We ran the regressions separately for each of the five vaccines (BCG, DPT1, DTP2, MCV1 and Pol3). Models were multivariable, using vaccination coverage as the dependent variable and the 11 factors as independent variables. The statistical analysis was performed using STATA version 15.

Next, we explicitly modelled countries as converging to steady-state outcome values to test conditional convergence in childhood immunization coverage [37, 38]. This conditional beta-convergence indicated the association between the average growth rate of a certain phenomenon (in this case, childhood immunization coverage) and its initial level. Therefore, we checked whether countries with similar structural parameters behaved more similarly to each other depending on the income group to which they belonged. For each country *i*, the average growth rate in outcome *Y* from period *t*−*τ* to period *t* would then depend on the difference between that country’s steady-state outcome value and its outcome value in period t−τ. The country’s steady-state outcome value was not observed but was allowed to vary by country and/or over time with the inclusion of country fixed effects and the socioeconomic factors considered in the analysis.

Therefore, to estimate first difference growth equations using lags as instruments for the endogenous covariates, we can write the convergence equation as follows:

$${cov}_{it}=(1+\beta ){cov}_{it-1}+X_{it}\delta +\alpha_{i}+\mu_{t}+\varepsilon_{it}$$ (1)

$$\Delta {cov}_{i}=\beta {cov}_{it-1}+X_{it}\delta +\alpha_{i}+\mu_{t}+\varepsilon_{it}$$ (2)

where *cov*_*it*_ is the vaccination coverage rate in country *i* at year *t*, *cov*_*it*−1_ is the lagged dependent variable; *α*_*i*_ is the country-fixed effects, *μ*_*t*_ is the year-fixed effects, *X*_*it*_ is a vector of control variables and *ε*_*it*_ is the error term.

If the coefficient (1+*β*) of the lagged dependent variable (i.e., delayed childhood vaccination coverage) is positive, there is evidence of conditional convergence. More specifically, this type of convergence exists when each country converges towards its own stationary state due to its own characteristics, which are very different from one another. In other words, each country can have its own stationary state due to the different characteristics that each one has, and they can converge towards their own stationary state in the long term, but never towards the same state.

## Results

**Fig 1:**
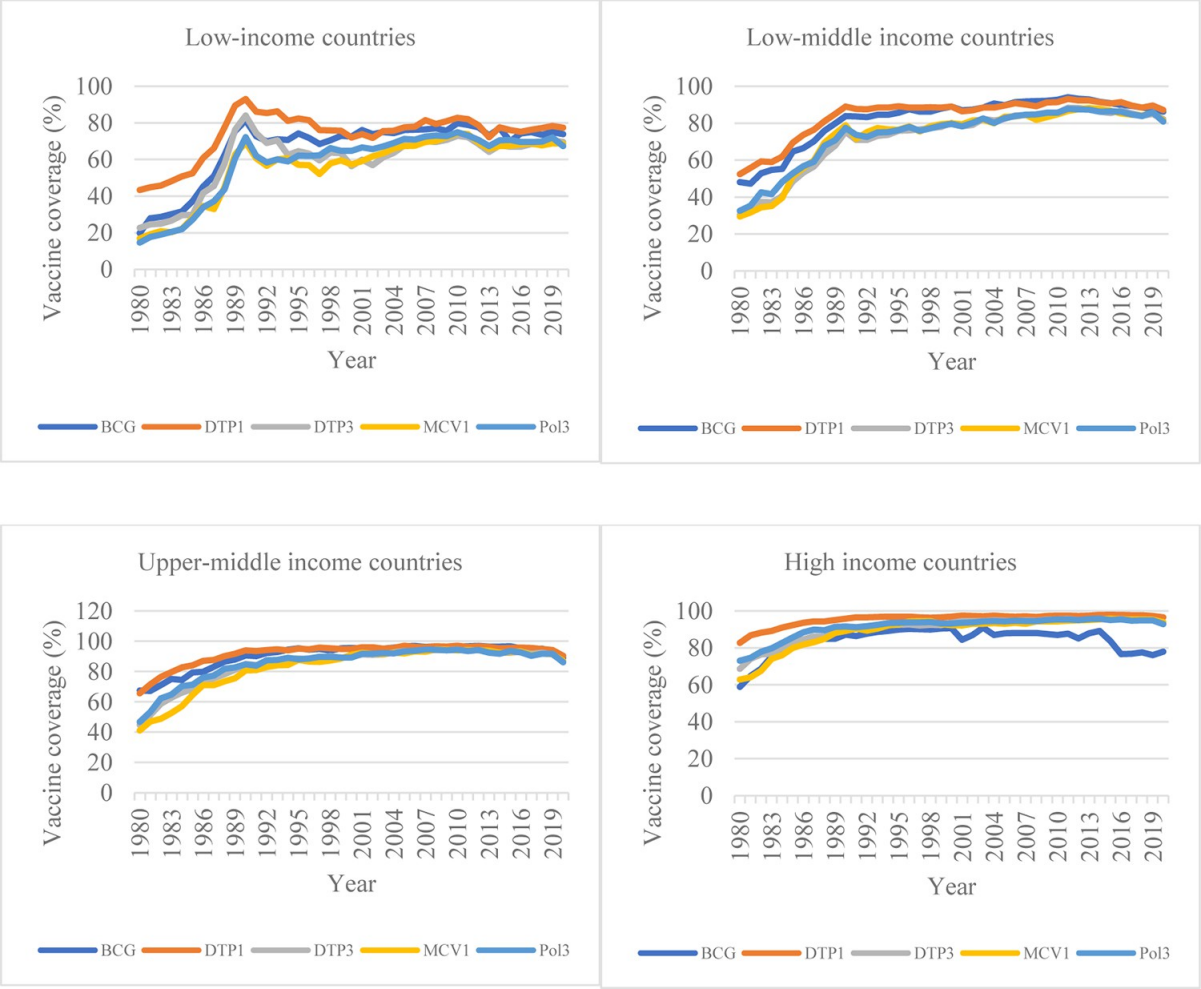
Trends in vaccination coverage in the four groups of countries analyzed by income over the period considered (1980–2020). Note: GNI per capita lower than $1,085 (low-income economies); GNI per capita between $1,086 and $4,255 (lower-middle income economies); GNI per capita between $4,256 and $13,205 (upper-middle income economies); GNI per capita of $13,205 or more (high-income economies). Source: Authors’ elaboration from the World Bank (2022).

Immunization rates have increased across the four income groups of countries over the period analyzed (from 1980 to 2020), as can be seen in Fig 1, where the trends in the average childhood vaccination coverage rates for the five vaccines analyzed are shown. It can be seen that each group of countries followed a similar trend. For example, the average vaccination rates increased in low-income countries between 1980 and 1990, after which they declined slightly until 1995. From then on, the average vaccination rates remained constant until the end of the period, reaching an approximate vaccination coverage of between 60% and 80%. Similarly, the average vaccination rates increased until 1990 in low-middle-income countries, after which they remained stable between 70% and 94%. In the case of upper-middle income economies, the average vaccination rates increased over the forty years considered, except in the last year (2020), when the rates of the five vaccines decreased slightly. The initial rates varied between 41% and 65%, while the end rates were around 90%. For high-income countries, the average vaccination rates increased between 1980 and 2020. All vaccines followed a similar trend, except BCG, the average vaccination rate of which started to decrease from 2000 until the end of the period.

Table 4 shows results from the GEE. In these models, factors used as independent predictors of immunization coverage were GDP per capita, current health expenditure, total population, population aged 65 and over, urban population, births attended by skilled health staff, hospital beds, nurses and midwives, physicians, surface area, and political stability and absence of violence/terrorism, which are shown in the first column. The results for each of the five vaccines considered as dependent variables are shown separately in columns 2–6, including the coefficient, standard deviation and p-value (the latter two in parentheses).

**Table 4:** Results of the Generalized Estimation Equations (GEE) (vaccination coverage rates as the dependent variable).

Variable	BCG Effect (std error; p-value)	DTP1 Effect (std error; p-value)	DTP3 Effect (std error; p-value)	MCV1 Effect (std error; p-value)	Pol3 Effect (std error; p-value)
GDP per capita	**7.416 (5.184; <0.0001)**	**10.086 (1.304; <0.0001)**	**7.539 (1.625; <0.0001)**	**12.068 (1.770; <0.0001)**	**8.488 (1.708; <0.0001)**
Current health expenditure	0.637 (0.847; 0.329)	**0.458 (0.215; 0.033)**	0.409 (0.263; 0.119)	**0.527 (0.267; 0.049)**	0.434 (0.276; 0.116)
Total population	4.106 (3.953; 0.299)	**4.589 (0.939; <0.0001)**	**3.869 (1.181; 0.001)**	**3.847 (1.342; 0.004)**	**4.667 (1.242; <0.0001)**
Population aged 65 and over	-0.796 (0.499; 0.105)	-0.210 (0.127; 0.099)	-0.201 (0.157; 0.199)	-0.301 (0.164; 0.067)	-0.260 (0.164; 0.113)
Urban population	0.039 (0.136; 0.905)	**-0.149 (0.033; <0.0001)**	**-0.114 (0.042; 0.006)**	**-0.098 (0.049; 0.046)**	**-0.108 (0.044; 0.014)**
Births attended by skilled health personnel	**0.433 (0.145; 0.003)**	**0.388 (0.038; <0.0001)**	**0.506 (0.048; <0.0001)**	**0.318 (0.051; <0.0001)**	**0.450 (0.050; <0.0001)**
Hospital beds	**2.982 (0.981; <0.0001)**	-0.172 (0.209; 0.412)	-0.122 (0.258; 0.636)	-0.259 (0.269; 0.335)	-0.203 (0.271; 0.454)
Nurses and midwives	0.115 (0.521; 0.558)	0.140 (0.127; 0.269)	-0.198 (0.154; 0.200)	-0.274 (0.155; 0.077)	-0.157 (0.162; 0.332)
Physicians	**-1.260 (0.914; <0.0001)**	**-1.389 (0.484; 0.004)**	-0.466 (0.599; 0.437)	0.115 (0.626; 0.854)	-0.425 (0.631; 0.500)
Surface area	-0.054 (3.438; 0.987)	-0.641 (0.787; 0.415)	-0.805 (1.011; 0.426)	-1.359 (1.225; 0.267)	-1.643 (1.064; 0.123)
Political stability and absence of violence/terrorism	-3.963 (2.099; 0.059)	0.520 (0.578; 0.369)	0.821 (0.703; 0.243)	0.310 (0.707; 0.661)	0.491 (0.739; 0.506)

Note: BCG (Bacillus Calmette-Guérin); DTP (Diphtheria, Pertussis and Tetanus); MCV (Measles-Containing vaccine); Pol (Polio). Statistically significant effects at the 0.05-level are shown in bold. Source: compiled by the author.

Starting with BCG, we found the strongest positive statistically significant association (at the conventional 0.05-level) with GDP per capita (coefficient of 7.416). The second strongest effect was that of hospital beds (2.982), followed by births attended by skilled health staff (0.433), which were both positively related as well. Conversely, the variable for physicians (-1.260) had a negative statistically significant effect.

With regard to DTP1, GDP per capita and births attended by skilled health staff had positive statistically significant effects (10.086 and 0.388, respectively), very similar to the effects we saw for DTP3 (7.539 and 0.506, respectively). Furthermore, total population exhibited a positive relation to DTP1 and DTP3 (4.589 and 3.869, respectively), while urban population (-0.149 and -0.114, respectively) had a negative association. For DTP1, we also saw statistically significant effects related to current health expenditure, which showed a positive coefficient (0.458), while the variable for physicians (-1.389) had a negative statistically significant effect.

In the case of MCV1, we found a positive statistically significant relationship between the vaccine coverage rate and GDP per capita (12.068), and births attended by skilled health staff (0.318). Moreover, total population (3.847) and current health expenditure (0.527) both showed a positive association, while urban population (-0.098) showed a negative relationship.

According to Pol3, we found the strongest positive statistically significant association with GDP per capita (8.488) and with births attended by skilled health staff (0.450), as happened with most of the vaccines analyzed. Furthermore, urban population (-0.108) had a negative association with Pol3, while total population (4.667) exhibited a strong positive, although much weaker, association.

**Fig 2:**
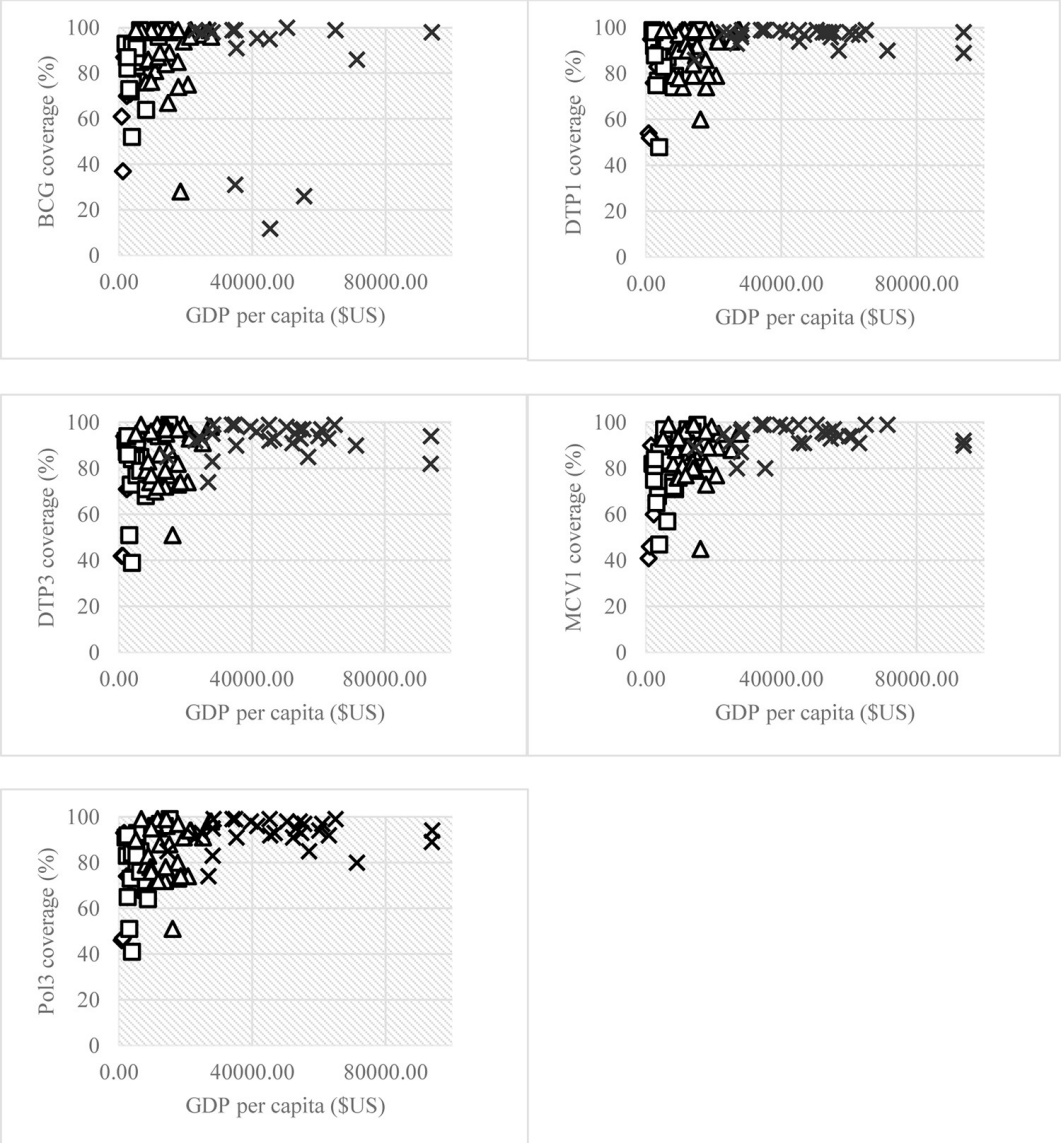
Scatter plot showing the association between the coverage of the five vaccines considered and GDP per capita in 2020. Note: Diamonds represent low-income countries, squares are lower-middle-income countries, triangles are upper-middle-income countries and crosses are high-income countries. GNI (Gross National Income). DTP1 and DTP3 are the first and the third dose of the vaccine for diphtheria, pertussis and tetanus; MCV1 is the first dose of the vaccine for Measles-Containing; Pol3 is the third dose of the polio vaccine. Source: Authors’ elaboration.

A scatter plot of the average coverage of the five vaccines finally selected (BCG, DTP1, DTP3, MCV1 and Pol3) against GDP per capita for the four country groups is shown below (see Fig 2). Most of the points are represented in the top left corner, indicating a general pattern of high vaccination coverage rates, although low-income countries had lower immunization rates. In addition, we found atypical data (outliers) when we focused on high-income economies. These data can be seen for the five vaccines, where the United Arab Emirates, Qatar and Ireland had a GDP per capita above $70,000 and vaccination rates that exceeded 80% coverage. However, we kept these countries in our sample after verifying that excluding them did not qualitatively modify our findings.

When we examined conditional convergence estimates (Table 5), we changed our dependent variable, i.e., the immunization coverage, for the average growth rate of immunization coverage and included the lagged vaccination rate. We ran five estimates again, one for each vaccine across all countries in the sample.

**Table 5:** Regression for conditional convergence, 1980–2020 (vaccination coverage rates as the dependent variable).

Variable	BCG	DTP1	DTP3	MCV1	Pol3
Lagged vaccination rate	**0.711 (0.052; <0.0001)**	**0.517 (0.048; <0.0001)**	**0.579 (0.044; <0.0001)**	**0.718 (0.037; <0.0001)**	**0.487 (0.046; <0.0001)**
GDP per capita	**8.001 (7.174; 0.026)**	**2.509 (1.638; 0.001)**	**3.134 (1.435; 0.029)**	3.172 (1.678; 0.059)	**3.395 (1.695; 0.045)**
Current health expenditure	**2.634 (1.201; 0.028)**	0.467 (0.274; 0.088)	**0.661 (0.254; 0.009)**	0.088 (0.241; 0.072)	0.522 (0.294; 0.076)
Total population	4.128 (7.518; 0.060)	2.386 (1.304; 0.067)	1.144 (1.214; 0.346)	**3.095 (0.990; 0.002)**	**3.627 (1.806; 0.045)**
Population aged 65 and above	**-2.011 (0.587; 0.001)**	-0.102 (0.200; 0.611)	**-0.405 (0.177; 0.022)**	**-0.398 (0.148; 0.007)**	-0.419 (0.213; 0.050)
Urban population	0.187 (0.134; 0.162)	**0.011 (0.039; 0.003)**	0.018 (0.048; 0.710)	0.027 (0.047; 0.572)	**0.234 (0.068; 0.001)**
Births attended by skilled health personnel	-0.223 (0.215; 0.080)	**0.212 (0.048; <0.0001)**	**0.138 (0.051; 0.006)**	0.036 (0.050; 0.471)	0.014 (0.062; 0.821)
Hospital beds	2.309 (1.318; 0.301)	0.026 (0.232; 0.909)	**-0.497 (0.253; 0.049)**	-0.226 (0.231; 0.328)	0.012 (0.243; 0.959)
Nurses and midwives	-0.539 (0.495; 0.276)	0.175(0.117; 0.136)	-0.111 (0.109; 0.311)	0.081 (0.108; 0.452)	-0.003 (0.122; 0.977)
Physicians	-1.146 (2.401; 0.633)	-0.828 (0.681; 0.224)	-0.502 (0.680; 0.461)	-0.336 (0.585; 0.566)	-0.358 (0.697; 0.608)
Surface area	**-2.230 (6.544; 0.002)**	-0.390 (1.043; 0.708)	1.010 (1.184; 0.394)	-1.769 (0.983; 0.072)	-2.365 (1.824; 0.195)
Political stability and absence of violence/terrorism	-3.901 (2.504; 0.119)	0.051 (0.619; 0.934)	**2.676 (0.716; <0.0001)**	0.237 (0.615; 0.700)	**1.698 (0.835; 0.042)**

Note: BCG (Bacillus Calmette-Guérin); DTP (Diphtheria, Pertussis and Tetanus); MCV (Measles-Containing vaccine); Pol (Polio). Statistically significant effects at the 0.05-level are shown in bold. Source: compiled by the author.

Our findings may validate the assumption of conditional convergence, since the lagged immunization coverage, which represents the relationship between starting vaccination coverage rate and its growth rate, is significantly positive for all the vaccines considered. In other words, the convergence path may be different in the range of countries analyzed, but introducing some key control factors, countries converge to their own steady state. In simple terms, this means that countries with lower (larger) initial levels of basic vaccination coverage are increasing (decreasing) coverage rates faster, depending on a number of country-specific characteristics.

We also added variables to account for the different economic, demographic, health, environmental, and political stability factors of immunization of the countries to observe how they may vary between the five vaccines considered. In this case, when control variables were not significant, they were unable to explain the process that led countries to converge towards different steady states. For example, GDP per capita was significantly positive in relation to the average growth rate of immunization coverage of all vaccines, except for MCV1. We found a positive relationship between vaccination coverage and current health expenditure for BCG and DTP3, as well as with total population for MCV1 and Pol3. Moreover, urban population exhibited a positive effect for both DTP1 and Pol3, while births attended by skilled health staff also did for DTP1 and DTP3. In the case of political stability and absence of violence/terrorism, there were positive associations for DTP3 and Pol3. Conversely, population aged 65 and over had a negative statistically significant effect for BCG, DTP3 and MCV1, as did hospital beds for DPT3 and surface area for BCG.

**Fig 3:**
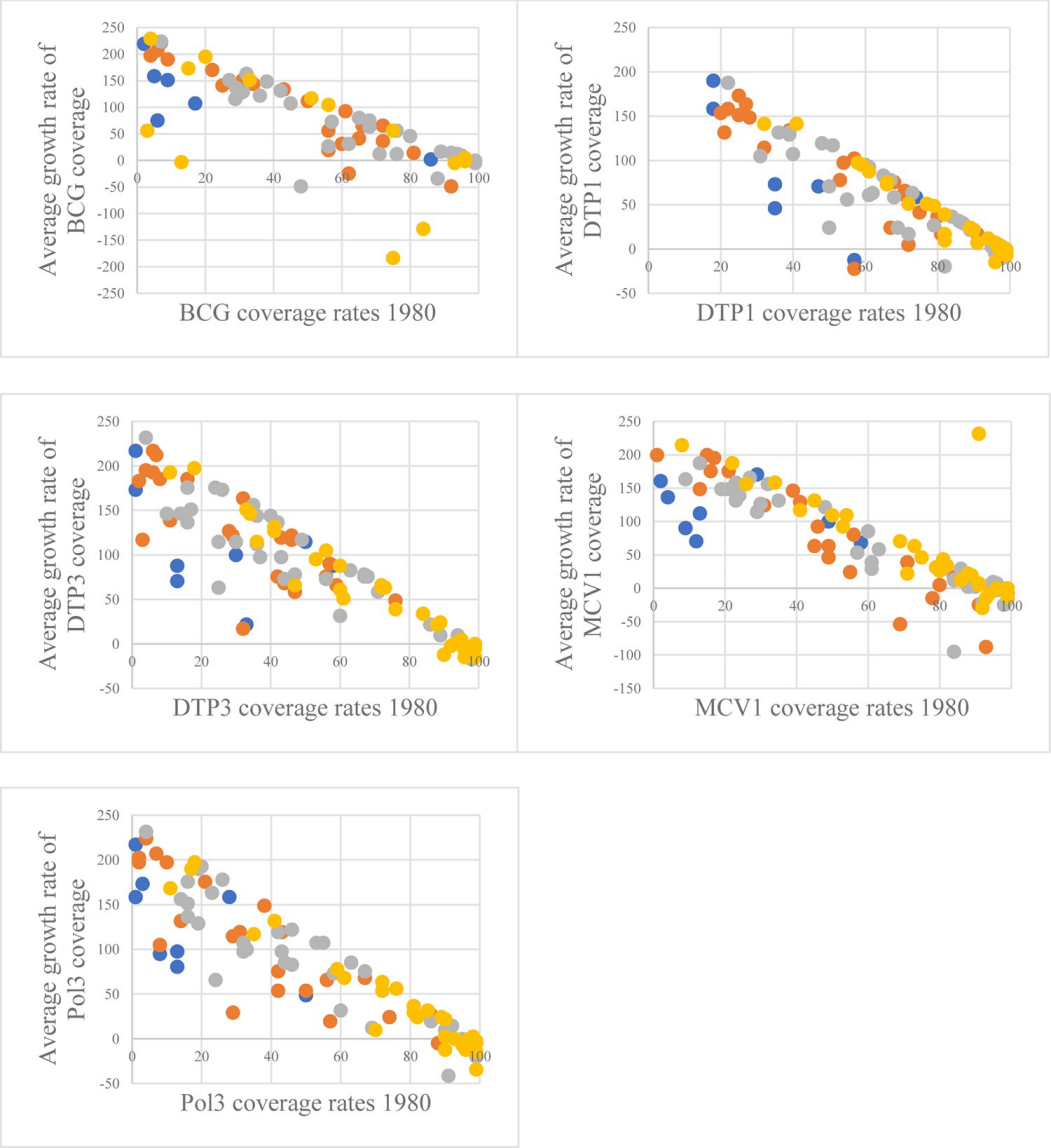
Conditional convergence for the five vaccines considered according to the World Bank classification. Note: The vertical line indicates the value for average real growth rate of each vaccine coverage, and the horizontal line indicates the value for initial vaccine coverage rates. Blue dots represent low-income countries, orange dots are lower-middle-income countries, grey dots are upper-middle-income countries and yellow dots are high-income countries. DTP1 and DTP3 are the first and the third dose of the vaccine for diphtheria, pertussis and tetanus; MCV1 is the first dose of the vaccine for Measles-Containing; Pol3 is the third dose of the polio vaccine. Source: Authors’ elaboration.

Furthermore, Fig 3 demonstrates that countries converge by income groups. As we examined countries based on the World Bank classification, we found that countries belonging to the same income group demonstrated similar behavior. This phenomenon is known as “convergence clubs”, which refers to the fact that different countries may have different types of equilibrium due to their initial characteristics. Consequently, underdeveloped countries and developed countries have their own conditional convergences.

## Discussion

We analyzed the association between basic vaccination coverage and national-level determinants related to economic, inequality, demographic, health, education, labor market, environmental, and political stability characteristics for five vaccines (BCG, DTP1, DTP3, MCV1 and Pol3) over the last four decades across 94 countries, which were divided into four groups based on World Bank classification (low-, lower-middle-, upper-middle- and high-income economies).

Previous literature has shown that immunization rates increase over time, especially for DTP and polio vaccines [24], but patterns of social inequalities in vaccine uptake (at least in developed countries) may differ depending on the context (vaccine, country …). In this sense, it is important to note that socioeconomic determinants explain inequalities in basic vaccination coverage.

National economic characteristics are important determinants, particularly GDP per capita, as it is often used as a positive indicator of the overall health of the population [39, 40] and it has a positive statistically significant relationship to immunization coverage [34]. In our analysis, GDP per capita was shown to be one of the strongest predictors of immunization coverage. In fact, a number of individual-level articles indicated that socioeconomic determinants play a more relevant role in vaccination coverage than other factors, such as negative parental beliefs, attitudes and perceptions for vaccinating children [41, 42]. In line with our results, previous studies have shown that expenditure on health has statistically significant positive effects [32, 43]. However, literature is not entirely conclusive when it comes to analyzing the association between vaccination and health expenditure, with some articles showing a negative relationship [34], with negative responses in vaccine importance, safety, effectiveness, and religious compatibility [39]. In this sense, independent variables of an economic nature may influence vaccination coverage, as the economic and financial prosperity of countries is expected to be associated with the success of their healthcare systems. There is a premise that richer countries have more resources to improve health compared to poorer countries, which are constrained by supply-side factors. Therefore, this has an impact on vaccination activities.

As far as demographic determinants are concerned, in line with other studies, our results further suggest that total population has a strong positive statistically significant effect for most vaccines. By contrast, population aged 65 and over shows negative coefficients. These findings are supported by other studies [34]. In the case of urban population, we found negative associations between immunization coverage and this demographic factor. Our conclusions were similar to previous reports [44], demonstrating through individual-level studies that children living in rural areas are more likely to be full vaccinated compared to urban areas [16, 45]. Nevertheless, other studies have not found such an association. Evidence has shown that the more children in urban areas, the higher the vaccination coverage rates [24, 26, 34]. In this context, lack of knowledge, level of education and misinformation can be factors that greatly affect the acceptance of vaccination by certain population groups. Governments need to pay greater attention to these determinants to reach a larger proportion of the population more efficiently and provide greater overall accessibility to vaccination.

We would also like to draw attention to the fact that health indicators may play potentially important roles in basic childhood immunization. Births attended by skilled health staff, which is considered to be maternal and postnatal care in our analysis, have strong positive contributions to childhood immunization coverage. This result is consistent with those of similar studies [32, 34]. This may partly reflect the development of health infrastructures and facilities, as well as logistics to facilitate current access to vaccination. Moreover, based on findings related to the density of health workers (nurses, midwives and physicians) and hospital beds, our results are not strongly associated with immunization rates, as is also the case in literature [43]. In addition, all these determinants showed negative effects for certain vaccines [34, 39]. The nature of this relationship is unclear. The lack of data collected in some of the 41 years analyzed and the imprecision in the measurement of the health worker densities could partly justify these weak associations.

The empirical evidence of this study revealed that environmental determinants, particularly surface area, showed statistically significant negative effects for all vaccines. These findings were in line with other similar studies, which show smaller areas as a relevant factor of vaccination [34, 43, 46]. This may suggest that, in large countries, in terms of land area, remote areas may present logistical difficulties in the provision of health services. In this sense, basic immunization of children may be more difficult to achieve in these areas compared to those in smaller countries.

In our analysis, political stability and absence of violence/terrorism showed significant positive effects on immunization rates for most vaccines (except BCG), probably reflecting the importance of political indicators on vaccination coverage levels. These findings are supported by other studies [34, 43, 46]. One possible explanation for these results could be general acceptance of and trust in the vaccination programs authorities have put in place, although the beliefs and attitudes of parents should not be forgotten, as these factors also play an important role. In other words, political instability, such as armed conflict, could entail population displacements and, therefore, vaccination logistics and access would be harder to maintain. This political situation would affect health by lowering the supply and demand for vaccines and, therefore, leading to lower childhood vaccination rates.

Our findings should be interpreted with caution in light of certain limitations. We considered an unbalanced panel data model due to the unavailability of data for some vaccines and/or socioeconomic determinants over the time horizon considered, although we used accepted international databases. Additionally, the selection of countries was determined by the availability of complete data for at least three of the five vaccines analyzed. Consequently, the sample consisted of a total of 94 countries. Future research using vaccination coverage data for all vaccines and countries considered are needed when such data are fully available. Next, as we used national childhood immunization coverage rates, we were unable to observe differences within countries or at a regional level. However, the macro-level results shown in this study extend the existing literature by analyzing basic vaccination coverage factors in the national context, since, as previously indicated, most of the published studies focus on the individual level. Moreover, some socioeconomic factors initially considered for the analysis had to be excluded because of a possible bi-directional causal relationship. In other words, life-expectancy at birth and under-5 mortality were rejected as health indicators. In the case of infant mortality, it cannot be considered exogenous, since it is a consequence (not a cause) of vaccination coverage in the inverse sense (negative sign of inverse causality: the more vaccination, the less infant mortality). In this sense, correlations were present in the analysis, given the number of variables considered. Considering the longitudinal nature of our data, we did not have the option to resolve multicollinearity using distances, partly because of the reduced interpretability of our results. For all of the above reasons, the results of this study should be treated with due caution.

## Conclusions

This paper provides new empirical evidence and insights, showing that several country-level predictors such as GDP per capita, health expenditure, total population, hospital beds, and births attended by skilled health staff made significant positive contributions to vaccination coverage rates, while urban population and density of physicians had negative significant associations. Moreover, the results also showed evidence of conditional convergence, which suggests that each country is converging towards its own steady state. In other words, countries are converging to their own level of vaccination coverage rate based on the World Bank classification (low, lower-middle, upper-middle and high-income economies), but conditionally on the different socioeconomic factors that exist between them.

Our findings may have significant implications in terms of formulating health policies to address basic childhood immunization coverage. Improving universal vaccination rates requires that the socioeconomic determinants mentioned above be taken into account in the design of programs to improve immunization status. Learning from country differences may help vaccine program managers and policymakers to understand the relative importance of the different factors that influence childhood vaccination coverage. Therefore, health policies are needed that are especially linked to national economic, demographic and health characteristics and aimed at reducing inequalities in childhood vaccination rates.

## Supporting information

S1 File Anonymized data set. (XLSX)
